# Decontamination of dental implant surfaces by means of photodynamic therapy

**DOI:** 10.1007/s10103-012-1148-6

**Published:** 2012-07-12

**Authors:** Juliana Marotti, Pedro Tortamano, Silvana Cai, Martha Simões Ribeiro, João Eduardo Miranda Franco, Tomie Toyota de Campos

**Affiliations:** 1 Department of Prosthodontics and Dental Materials, Medical Faculty, University Hospital RWTH Aachen, Pauwelsstrasse 30, 52074 Aachen, Germany; 2 Department of Prosthodontics, School of Dentistry, University of São Paulo, Av. Prof. Lineu Prestes, 2227, 05508-000 São Paulo, SP Brazil; 3 Department of Microbiology, Biomedical Institute, University of São Paulo, Av. Prof. Lineu Prestes, 2415, 05508-000 São Paulo, SP Brazil; 4 Center for Lasers and Applications, IPEN-CNEN/SP, University of São Paulo, Av. Prof. Lineu Prestes, 2242, 05508-000 São Paulo, SP Brazil; 5 Departamento de Prótese, Faculdade de Odontologia, Universidade de São Paulo, Av. Prof. Lineu Prestes, 2227, 05508-000 São Paulo, SP Brazil

**Keywords:** Decontamination, Dental implantation, Lasers, Methylene blue, Photodynamic therapy, Titanium

## Abstract

Several implant surface debridement methods have been reported for the treatment of peri-implantitis, however, some of them can damage the implant surface or promote bacterial resistance. Photodynamic therapy (PDT) is a new treatment option for peri-implantitis. The aim of this in vitro study was to analyze implant surface decontamination by means of PDT. Sixty implants were equally distributed (*n* = 10) into four groups and two subgroups. In group G1 there was no decontamination, while in G2 decontamination was performed with chlorhexidine. G3 (PDT − laser + dye) and G4 (laser, without dye) were divided into two subgroups each; with PDT performed for 3 min in G3a and G4a, and for 5 min in G3b and G4b. After 5 min in contact with methylene blue dye (G3), the implants were irradiated (G3 and G4) with a low-level laser (GaAlAs, 660 nm, 30 mW) for 3 or 5 min (7.2 and 12 J). After the dilutions, culture media were kept in an anaerobic atmosphere for 1 week, and then colony forming units were counted. There was a significant difference (*p* < 0.001) between G1 and the other groups, and between G4 in comparison with G2 and G3. Better decontamination was obtained in G2 and G3, with no statistically significant difference between them. The results of this study suggest that photodynamic therapy can be considered an efficient method for reducing bacteria on implant surfaces, whereas laser irradiation without dye was less efficient than PDT.

## Introduction

At the Sixth European Workshop on Periodontology, peri-implantitis was described as an inflammatory process affecting the tissues around an osseointegrated implant, associated with suppuration, deepened pockets, and loss of supporting marginal bone [1]. Successful treatment of peri-implantitis continues to be challenging because of its complexity. During the surgical stage, the steps involved include the elimination of plaque and calculus, decontamination of the implant surface, guided tissue regeneration, and finally, maintenance of healthy conditions [2].

Effective decontamination of dental implant surfaces is one of the most difficult steps; and for this reason, several different treatments have been proposed in the literature [3–8]. Titanium implant surfaces can be cleaned by mechanical means (dental curettes, ultrasonic scalers, air–powder abrasive) and/or chemical procedures (citric acid, H_2_O_2_, chlorhexidine digluconate, and EDTA), usually associated with local or systemic antibiotics [9–12]. However, some of these methods can damage the surface properties of implants or promote bacterial resistance [13–15].

Recent studies have demonstrated that use of lasers can be helpful in decontamination of titanium implants. The lasers most frequently used in peri-implant care include CO_2_, diode, and erbium lasers, due to their hemostatic properties, selective calculus ablation and bactericidal effects. However, high power lasers can promote an undesirable increase in temperature. Another disadvantage is the high cost of equipment [16–19].

A potential alternative approach to dental implant decontamination is the association of the conventional treatment with photodynamic therapy (PDT). PDT can be described as the association of light with a suitable photosensitizer in the presence of oxygen. It is based on the principle that a photosensitizer binds to the target cells and when it is irradiated with light of specific wavelength, in the presence of oxygen, it undergoes a transition from a low-energy ground state to an excited singlet state, then singlet oxygen and other very reactive agents are produced, which are toxic to these target cells [19, 20].

Furthermore, it seems unlikely that resistance to PDT will develop, since its bactericidal activity is due to singlet oxygen and other reactive species such as hydroxyl radicals, which affect a range of cellular targets [21]. Many studies have demonstrated that lethal photosensitization of bacteria can be achieved in vitro without any damage to the treated titanium surfaces [20, 22–24].

Although studies have been conducted on the decontamination of dental implant surfaces by means of PDT, there is still no consensus in the literature about which PDT irradiation parameter would be best for bacterial reduction. Thus, the aim of this in vitro study was to analyze the bacterial decontamination of dental implant surfaces by means of photodynamic therapy, using two different irradiation times, in order to create conditions for a further in vivo study.

## Materials and methods

This study was approved by the Research Ethics Committee of the School of Dentistry, University of São Paulo (USP), Protocol #68/2008.

### Groups

Anodized implants with rough surfaces (TiUnite, Nobel, 12 × 4 mm) were used, *n* = 60, which were equally divided into two groups and two subgroups, *n* = 10 for each group (Table 1). G1 and G2 were the control groups. All groups were contaminated. In G1, no decontamination was performed (negative control), while in G2 (positive control) decontamination was performed by the traditional method using a 0.12 % chlorhexidine gluconate solution (PerioGard, Colgate-Palmolive). Group G3 was decontaminated by PDT (dye + laser). In group G4, laser irradiation was used, however, without dye application, in order to better evaluate the effectiveness of the use of dye on the action of PDT. Groups G3 and G4 were subdivided into two subgroups, with irradiation being performed for 3 min in G3a and G4a, and for 5 min in G3b and G4b.

**Table 1:** Distribution of experimental and control groups

Groups	Decontamination method	
G1 (negative control)	contaminated, not decontaminated (*n* = 10)	
G2 (positive control)	0.12 % Chlorhexidine (*n* = 10)	
G3 (PDT − laser + dye)	(a) Laser 3 min (*n* = 10)	(b) Laser 5 min (*n* = 10)
G4 (without dye, with laser)	(a) Laser 3 min (*n* = 10)	(b) Laser 5 min (*n* = 10)

### Manipulation

The brand new implants were carefully removed from the cases provided by the manufactor, placed on a black plate and manipulated with titanium implant plier, when necessary. All materials were sterile.

To contaminate the implants, 30 mL of saliva was collected from a patient previously diagnosed with peri-implantitis in four implants, and the implants were kept in this saliva for 5 min. Immediately afterwards, the decontamination procedure was performed. No drying time was allowed, so all implant surfaces were treated wet.

All the steps in the methodology of this study were performed at the laboratory of the Biomedical Institute of the University of São Paulo, São Paulo, Brazil, under the same conditions (22 °C, 60 % humidity, <1 % CO_2_, 925.5 hPa).

### Decontamination

The implants were immersed in 3 mL chlorhexidine solution (G2) or in 3 mL of the methylene blue dye (Chimiolux–Hypofarma, Belo Horizonte, MG, Brazil), at a concentration of 0.01 % (mass per volume) for 5 min. The dye was used only in group G3 and after 5 min, laser irradiation was performed with an GaAlAs low-level diode laser (Twin Laser Flex, MM Optics—São Carlos, Brazil), at a wavelength of 660 nm. The output power of 30 mW was previously checked with a power meter (Power Meter 841-PE, Newport Corporation, USA) using a specific PDT fiber optic (∅ 0.5 mm, 50 mm length, MM Optics). Group G4 received the same treatment as G3, however, without the dye.

**Fig. 1:**
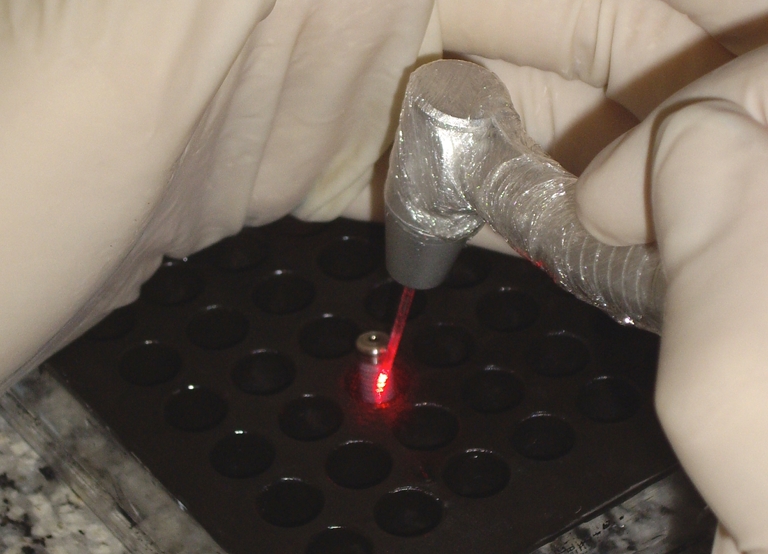
Irradiation on the surface of the implant placed on the black colored prefabricated plate

The irradiation time was 3 or 5 min, according to the subgroups, and the total energy released was 7.2 J for the time of 3 min, and 12 J for 5 min. The fiber optic was developed especially for use in PDT. Irradiation over the entire exposed external surface of the implant was performed in contact mode. The fiber optic was positioned at one point, then in another point, and so on successively, until the irradiation time was reached (Fig. 1).

After decontamination with PDT or chlorhexidine solution, the implants were gently and slowly irrigated with two syringes, each containing 3 mL of sterile physiological solution. This was so that the remainders of chemical substances would not be transported to the culture medium, which would harm colony growth, particularly in the case of chlorhexidine. For the purpose of standardization, all groups were equally irrigated with sterile physiological solution, by the same calibrated operator, before microbiological analysis.

### Analysis of decontamination

Bacterial decontamination was quantitatively analyzed by seeding saliva in a culture medium and then counting colony forming units. This was performed using a stereoscopic microscope (Bausch & Lomb), at ×10 magnification. After the different groups were decontaminated, the implants were placed in previously sterilized microcentrifugal tubes containing peptonized water, and agitated for 30 s, in order to detach the bacteria. Serial dilutions were then made and aliquots of 20 μL were dripped on Brucella agar, according to the Miles and Misra method [25]. For each dilution, three drops were seeded. The plates were incubated at 37 °C for 7 days in an anaerobic atmosphere. The number of bacteria per milliliter was calculated according to the following formula: no. of bacteria per milliliter = mean no. of colonies in the three dilutions × 1/dilution × 50.

Of the 10 implants evaluated in each group, three samples (three drops) were collected for the diluted solution of each implant. Initially, descriptive statistics were calculated; that is, mean, standard deviation, minimum, maximum, and median values of data obtained from each of the collections made for each group. Afterwards, the three collections were united and summed up by the arithmetical mean, and the summarized statistics of each group were calculated again. Table 2 shows the summarized measures of the number of bacteria per group.

**Table 2:** Summarized measures of the number of bacteria (×10^3^)/mL, per group

Group	*N*	Mean	SD	Minimum	Median	Maximum
G1	10	483	448	0	333	1167
G2	10	0.622	1.070	0.000	0.158	2.900
G3a	10	1.772	1.310	0.167	1.500	3.633
G3b	10	0.603	0.995	0.017	0.225	3.183
G4a	10	11.550	7.750	4.000	9.170	25.830
G4b	10	10.590	10.590	1.170	10.580	39.330

*N* number of implants evaluated per group, *SD* standard deviation

All the analyses were performed with the use of the Minitab version 15.0 statistical software. The level of significance was established at 5 % (*α* = 0.05), for the overall comparisons (all the groups), or 0.5 % (*α* = 0.005) in the individual comparisons between two groups, using the Kruskal–Wallis test. The normality was observed by the Kolmogorov–Smirnov test (*p* < 0.01) and the difference between groups by the Mann–Whitney test.

## Results

**Fig. 2:**
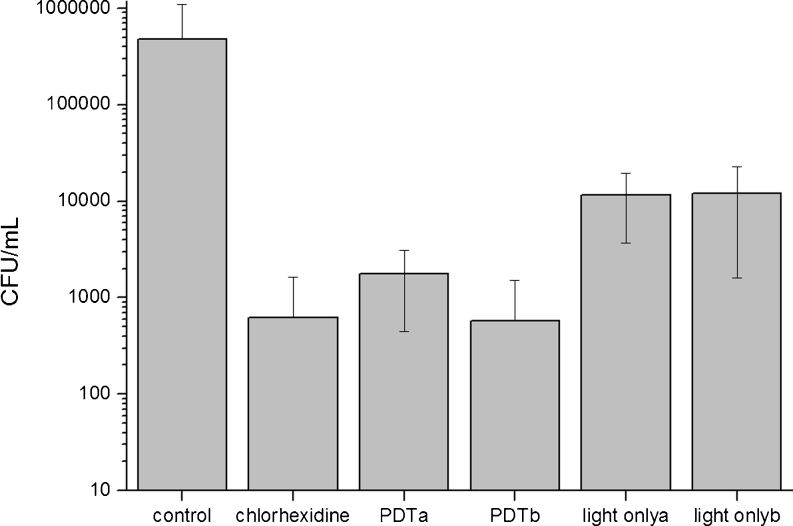
Comparison of all groups after decontamination, given in log10 scale and standard deviation

The data are presented in Fig. 2, showing the great difference in the number of bacteria found on the implants from G1, in comparison with the other groups (statistically verified by the Kolmogorov–Smirnov normality test, *p* < 0.010). Therefore, comparison among the groups was made by the nonparametric Kruskal–Wallis test, after dispensing with the supposition of data normality.

When the Kruskal–Wallis test was applied, statistical significance was found (*p* < 0.001), indicating that there was, in fact, significant difference among groups G2, G3a, G3b, G4a, and G4b. To find out exactly which group differed from which, the Mann–Whitney test was used to make comparisons between two groups. Table 3 shows the results of the Mann–Whitney test.

**Table 3:** Descriptive level (*p* value) of the Mann–Whitney test for comparison between two groups

	G2	G3a	G3b	G4a	G4b
G2	–	0.154	0.649	*<0.001*	*<0.001*
G3a	0.154	–	0.011	*<0.001*	*0.002*
G3b	0.649	0.011	–	*<0.001*	*<0.001*
G4a	*<0.001*	*<0.001*	*<0.001*	–	0.999
G4b	*<0.001*	*0.002*	*<0.001*	0.999	–

As described in the statistical methodology, with the level of significance adjusted to *α* = 0.005 for all the multiple comparisons, it was concluded that groups G2, G3a, and G3b presented lower levels of contamination than groups G4a and G4b, and there was no significant difference among G2, G3a, and G3b. There was also no significant difference between groups G4a and G4b.

**Fig. 3:**
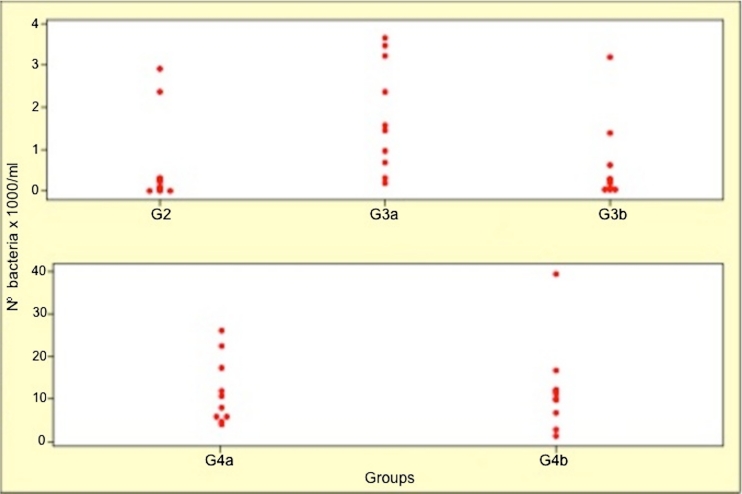
Point graph: no of bacteria (×10^3^)/mL, per group (except G1)

Figure 3 shows all the measurements obtained, indicating similarity among groups G2, G3a, and G3b, and also between groups G4a and G4b. G1 is not represented because of its great discrepancy in relation to the other groups, so a better relationship between G2, G3, and G4 can be seen.

## Discussion

Although the photodynamic therapy was first used in Medicine over a 100 years ago for cancer treatment [26], only recently has antimicrobial photodynamic therapy been introduced in dentistry [20]. The benefits of laser and PDT in the many specialties of dentistry have been described in the literature, but only in the last few decades has there been increasing interest by the scientific community as regards the benefits of PDT in implant dentistry, and as a coadjuvant treatment for peri-implantitis [27–30]. Branemark’s discovery of osseointegration in 1965 was extremely important to esthetic prosthetic restorative treatments and particularly, functional oral rehabilitation. An increasing number of patients have been rehabilitated with dental implants, and consequently, more cases of success and failure have appeared over the years. Thus, peri-implantitis has become an increasingly frequent problem in dental clinics [31].

In this study, the implants were contaminated with saliva collected from a patient previously diagnosed with peri-implantitis. This patient had a history of periodontitis and he was under control treatment. Given the similarities between the diseases processes of periodontitis and peri-implantitis, patients with a history of periodontal disease may be more susceptible to peri-implantitis, and this hypothesis has been supported by increasing evidence [32, 33]. In partially edentulous patients, periodontal pathogens may be transmitted by saliva from periodontally compromised teeth to newly placed implants [34]. Thus, it is important to treat periodontitis before dental implant placement [34, 35].

Different therapies have been proposed in the literature, with the aim of decontaminating the implant surface, however, none of them have been satisfactory up to now. PDT appears as another option for bacterial reduction; nevertheless, there is still no ideal protocol. Based on this doubt, the aim of the present study was to evaluate only a few of the parameters of this complex therapy.

The results obtained were in agreement with those that were expected, based on previous studies in the literature [27–30, 36, 37]. As group G1 was the negative control, in which no decontamination technique was used, it was expected to observe a great difference in the number of bacteria, in comparison with other groups. It was also expected that a larger number of bacteria would be found for Groups G4a and G4b, in comparison with the chlorhexidine (G2) and PDT (G3a and G3b) groups, as did in fact occur.

One may ask whether bacterial adherence was promoted. In fact, 5 min of implant contact with the contaminated saliva may not have been sufficient time for bacterial adherence. However, the authors were not concerned about having effective adherence, but rather to have enough time to ensure the presence of bacteria on the implant surface; which justifies the use of the term decontamination that is valid for both situations, i.e., whether microorganisms are attached or not. Due to the rough implant surface, indeed most of the bacterial were still attached to it, in spite of the irrigation with saline solution, which explains the great difference between G1, in which no decontamination process was performed, and the other groups.

A great difference was noted between G1 and G4. A possible reduction in the number of bacteria in G4, in comparison with G1, was caused by the absorption of light by pigmented bacteria that have endogenous chromophores, which dispenses the use of an additional photosensitizing agent, thus the effects of photodynamic therapy also occurred. According to König et al. [38], bacteria such as *Porphyromonas gingivalis*, *Prevotella intermedia*, and *Actinomyces odontolyticus* are capable of synthesizing protoporphyrin and proto-hematoporphyrin, one of the most frequently used dyes in photodynamic therapy, which does not require the additional use of external photosensitizers. Thus, mere irradiation with red-emitting laser produces the death of these microorganisms. Through microscopic observation, the pigmented bacteria could be seen in this study, however, it cannot be affirmed which species were present, since our methodology evaluated the population of bacteria and not the specific types.

When group G4 was compared with G3, it was observed that the use of dye was important in achieving greater bacterial reduction, and this difference was statistically significant (*p* < 0.001). This result proves the real effectiveness of the association of dye with the laser light source.

There was no significant difference between G4a and G4b; therefore, the irradiation time without the presence of dye did not interfere in bacterial reduction, which did not occur when G3a and G3b were compared.

In a future study, it would be interesting to increase the time of dye contact with the implant, and the irradiation time. A longer time in contact with the photosensitizer could allow more bacteria to be affected, particularly the Gram-negative type, which are more resistant to dye penetration due to the presence of an external membrane.

The results of this study complement the theory of the effectiveness of the association of laser + photosensitizer in previous studies [36, 39–42] when groups G3 and G4 are compared. In this study, while it was observed that irradiation with laser only was significantly less effective than photodynamic therapy, Chan and Lai [36] proved that laser only, or 0.01 % methylene blue only had no toxicity against bacteria. The authors also stated that the dye did not convert laser energy into heat, proving that the decontamination was not due to a possible excessive increase in intracellular temperature. Dobson and Wilson [39] and Prates et al. [40] also stated that cell death was not significant in the presence of dye only or laser only.

The best results were obtained in groups G2 and G3 (chlorhexidine and PDT, respectively), differing statistically in comparison with G1 and G4 (*p* < 0.001); however, there was no statistically significant difference between G2 and G3. Very similar results were observed between G2 and G3b (PDT 5 min). It is known that chlorhexidine has the capacity to be gradually released, and can act in periods of up to 24 h in vivo. Although the implants were abundantly irrigated with physiologic solution after decontamination with chlorhexidine, rests of the chemical substance may have remained on the implant surfaces, and consequently, have been transported into the culture medium, and continued to act against the bacteria.

Differences in the microbial analysis results with regard to the use of chlorhexidine are mainly due to the evaluation methodology and the diffusion of chlorhexidine into agar [43]. Whether or not this hypothesis is considered, the fact that G3 was statistically equal to G2, allows one to consider that photodynamic therapy would, nevertheless, be a more indicated method for decontaminating the surface of implants than irrigation with chlorhexidine solution, as it does not cause bacterial resistance and has the additional benefit of laser irradiation. The light that was not absorbed by the bacteria could be scattered and absorbed by chromophores of the adjacent peri-implant tissue, promoting biomodulation of the tissues (analgesic effect, modulation of inflammation, acceleration of the gingival and bone tissue repair processes, etc.) [44–47].

The results obtained in this study suggest that photodynamic therapy could be considered an effective method for bacterial reduction on implant surfaces and that laser irradiation alone, without the association of dye, was less efficient (*p* < 0.001) than PDT. Photodynamic therapy should, however, be considered a coadjuvant in the treatment of peri-implantitis and associated with mechanical (scaling) and surgical (grafts) treatments in an endeavor to control peri-implant disease.

Further studies should be conducted to test not only the laser irradiation parameters, but the photosensitizer agent, time of permanence, application mode (solution, paste, etc.), concentration, among other variables, so that an ideal protocol for the use of photodynamic therapy for the treatment of peri-implantitis may be achieved.

## Conclusions

Within the parameters used in this study, it could be concluded that photodynamic therapy can be considered an efficient method for reducing bacteria on implant surfaces. Laser irradiation alone, without the association of dye, was less efficient than phtodynamic therapy.
